# Schisandrin B downregulates exosomal fibronectin 1 expression to inhibit hepatocellular carcinoma growth

**DOI:** 10.3389/fphar.2025.1547685

**Published:** 2025-03-28

**Authors:** Baoyi Jiang, Jie Yang, Qingtian Huang, Wei Li, Qian Peng, Huoye Gan, Tieli Peng, Leyi Yao, Ling Qi

**Affiliations:** ^1^ Division of Gastroenterology, Institute of Digestive Disease, The Affiliated Qingyuan Hospital (Qingyuan People’s Hospital), Guangzhou Medical University, Qingyuan, Guang Dong, China; ^2^ Department of Pathology, The Affiliated Qingyuan Hospital, Guangzhou Medical University, Qingyuan People’s Hospital, Qingyuan, Guang Dong, China; ^3^ Biological Sample Resource Centre, The Affiliated Qingyuan Hospital, Guangzhou Medical University, Qingyuan People’s Hospital, Qingyuan, Guang Dong, China; ^4^ Zhanjiang Institute of Clinical Medicine, Zhanjiang Central Hospital, Guangdong Medical University, Zhanjiang, China

**Keywords:** Schisandrin B, hepatocellular carcinoma, exosomes, fibronectin 1, tumor microenvironment

## Abstract

**Introduction:**

In recent years, natural compounds have attracted wide attention for the treatment of liver cancer due to their therapeutic potential and reduced toxicity. Among these, Schisandrin B (Sch B), a primary bioactive component derived from Schisandra chinensis, has shown notable antitumor activity; however, its specific mechanism remains unclear.

**Methods:**

The effect of Sch B on the growth of hepatocellular carcinoma(HCC) cells were assessed using CCK-8 assay, colony formation assay and EdU assay, and apoptosis was detected by flow cytometry. The co-culture system of macrophages and HCC cells was established to detect the effect of Sch B on the cell viability and cell cycle changes of HCC cells in the co-culture system. Then, the migration of HCC cells in the co-culture system was studied using a subtoxic concentration of Sch B. Exosomes of the co-culture system with or without Sch B effect were collected for identification and protein spectrum analysis. The differential protein was analyzed by KEGG enrichment analysis and protein interaction network, which was verified by western blotting. Meanwhile, the expression changes of macrophage polarization markers were detected. Finally, the inhibitory effect of Sch B on HCC and the changes of FN1 were verified by in vivo experiments.

**Results:**

Sch B inhibited HCC cell growth; moreover, it significantly suppressed HCC cell proliferation in the co-culture system and induced S-phase cell cycle arrest by downregulating CDK4, CDK2, and cyclin A2 while upregulating p27 Kip1. Additionally, Sch B inhibited the migration of HCC cells in the co-culture system.The differentially expressed protein fibronectin 1(FN1) in liver cancer patients was higher than that in healthy people. Moreover, after SchB treatment, the expression of FN1 protein in exosomes decreased and the macrophages exhibited M1 polarization. In vivo experiments also verified that Sch B inhibited HCC growth and downregulated the expression of FN1 protein in tumor tissues.

**Conclusion:**

Sch B may inhibit the development of HCC by inhibiting the expression of exosomal FN1during interactions between macrophages and HCC cells.

## Introduction

Cancer incidence and mortality rates continue to increase, exacerbating the global healthcare burden (Sun et al., 2020). Primary liver cancer is one of the most common malignant tumors of the digestive system worldwide and is the third leading cause of cancer-related deaths in 2022 (Bray et al., 2024). Hepatocellular carcinoma (HCC) is the primary type of primary liver cancer, accounting for 90% of all cases. It is highly aggressive and has a low survival rate (Llovet et al., 2021). Patients with liver cancer are typically diagnosed in the middle and late stages of the disease, and few treatment options are available. Sorafenib has been shown to be effective in patients with severe cirrhosis who are not eligible for liver-directed treatment and in patients with metastatic HCC with slow disease progression. However, some patients are still insensitive to sorafenib (Saffo and Taddei, 2019). Advanced HCC can be treated with chemotherapy, immunotherapy, and oncolytic viruses. The combined use of chemotherapy and immunotherapy can further improve treatment effect (Alawyia and Constantinou, 2023). Despite this, the high mortality rate of liver cancer indicates that the current treatment options for HCC have not achieved the expected treatment goals. The tumor microenvironment (TME) is a key pathological environment in the development of HCC. Multiple components coexist and interact with the TME. In addition to tumor cells, macrophages are important components of the TME and are involved in cancer progression (Tian et al., 2019). Macrophages are the most abundant immune cells in the liver and play a key role in maintaining liver homeostasis and understanding the underlying mechanisms of liver disease (Wen et al., 2021). They are also the most abundant immune cells that infiltrate liver cancer cells and actively participate in tumor-specific inflammation and immunosuppression. In addition to direct contact between cells, cell interactions and communication can also promote signal transduction between cells by secreting factors. Exosomes play an important role in mediating the secretion of these factors. As an emerging component of tumor-host interactions, exosomes are increasingly recognized as information transfer vectors in the TME and as key molecular entities involved in the construction of tumorigenic microenvironments (Wu et al., 2019).

Exosomes are extracellular vesicles with diameter of 30–150 nm that can be secreted and released by all living cells and are loaded with a variety of bioactive molecules, including nucleic acids, proteins, lipids, and metabolites (Yokoi and Ochiya, 2021). They can transport bioactive molecules from near to far, or into body fluids to bridge the communication of signals between cells. For example, tumor-derived exosomes can promote the metastasis of colorectal cancer to the liver by regulating the interaction between colorectal cancer cells and tumor-associated macrophages (TAMs) (Zhao et al., 2020). They also deliver tumor suppressor factors that induce M2 macrophage polarization, thereby promoting glioma progression (Li et al., 2022). Therefore, exploring the mechanism of action of exosomes in the liver cancer microenvironment could provide a new therapeutic strategy for the treatment of liver cancer.

Current specific drugs for HCC are often accompanied by side effects (such as nausea and vomiting), whereas natural compounds exhibit structural similarity to chemical compounds and show similar anti-cancer potential, low toxicity and no adverse reactions. Schisandrin B (Sch B) is the most active dibenzocyclooctadiene derivative extracted from the Chinese herbal medicine *Schisandra chinensis* and is the primary chemical component of *S. chinensis* lignans (Li S. et al., 2021; Li Z. et al., 2021). An increasing number of studies have shown that Sch B has multiple effects, including anti-asthma, antioxidant, anti-inflammatory, and anticancer effects (Chen et al., 2021; Nasser et al., 2019). In the liver, Sch B can improve liver damage by inducing autophagy (Li et al., 2023) and reduce liver fibrosis by attenuating hepatic stellate cell activation and promoting cell apoptosis (Li Z. et al., 2021). In addition, Sch B can promote the pyrodeath of HCC cells by activating NK cells, thus playing an anti-tumor role (Song et al., 2023). Macrophages are important components of the TME, and exosomes are important signaling molecules for intercellular communication. Therefore, whether Sch B can affect the interaction between liver cancer cells and macrophages in the TME to affect the occurrence and development of liver cancer remains unclear. This study explored from the mechanism from the perspective of exosomes, finding that Sch B can inhibit the growth of HCC cells in the interaction system, which may be mediated through regulating FN1 expression in the TME.

## STAR methods

### Cell culture and drug treatment

HCC cell lines HepG2 and HCCLM3, and THP-1 were purchased from ATCC. HCC cells were cultured in high-glucose Dulbecco’s modified Eagle medium (DMEM) supplemented with 10% fetal bovine serum (FBS), whereas THP-1 cells were cultured in RPMI-1640 medium containing 10% FBS. THP-1 cells were treated with 100 ng/mL PMA (ACMEC) for 48 h to induce macrophages differentiation. These cells were cultured in an incubator at 37°C and 5% CO_2_.

Dimethylsulfoxide (DMSO) was used as a solvent to dissolve the Sch B powder (Selleck, S3600). The Sch B stock solution was diluted 1,000 times for use. An equal volume of DMSO was added to the control group. The final DMSO concentration did not exceed 1/1,000.

### Establishment of co-culture system

After thoroughly washing the PMA-treated THP-1 macrophages (upper chamber), they were co-cultured with HCC cells (in a six-well plate) without direct contact. Sch B was added for 48 h, the upper chamber containing macrophages was discarded, and the HCC cells were used for subsequent experiments.

### Cell proliferation assay

The viability of cells treated with Sch B for 48 h was determined using the Cell-Counting Kit (CCK)-8 kit (GLPBIO, CK04). Cell proliferation was detected using an EdU cell proliferation detection kit (RIBOBIO, Guangzhou, China, C10310). In the colony formation laboratory, HCC cells were seeded into six-well plates at a density of 800 cells/well. After adding different concentrations of Sch B for 48 h, the culture medium was replaced with fresh medium, and the cells were cultured for 14 d. Colonies were fixed with formaldehyde, stained with 1% crystal violet, counted, and quantified.

### Western blotting assay to detect protein expression

Cells and exosomes were lysed with RIPA lysis buffer (Solarbio, Beijing, China, R0010) to extract total protein. Protein concentration was determing using a bicinchoninic acid (BCA) kit (Solarbio, Beijing, China, PC0020), and denatured protein samples were separated using 10% sodium dodecyl-sulfate polyacrylamide gel electrophoresis (SDS-PAGE) and transferred to a PVDF membrane. The membrane was blocked with 5% milk for 1 h, incubated with the corresponding primary antibody overnight, and then incubated with a horseradish peroxidase-conjugated secondary antibody for 1 h. The intensity of protein bands was determined using a Bio-Rad system (Bio-Rad Laboratories, Hercules, CA, United States). GAPDH (1:1,000, 2,118), Alix (1:1,000, 2,171), β-actin (1:1,000, 4,970), CDK2 (1:1,000, 18,048), CDK4 (1:1,000, 12,790), cyclin A2 (1:1,000, 67,955) and p27 Kip1 (1:1,000, 3,686) were purchased from Cell Signaling Technology. Antibodies against CD9 (1:1,000, ab223052) and fibronectin 1 (FN1) (1:1,000, ab268020) were purchased from Abcam (Cambridge, United Kingdom).

### Quantitative real-time PCR

Total RNA of M0 macrophages in co-culture system was extracted using Trizol (Invitrogen) and reverse transcription was performed with the HiScript III All-in-one RT SuperMix Perfect for qPCR (Vazyme). Specific quantitative real-time PCR experiments were performed using the Taq Pro Universal SYBR qPCR Master Mix, according to the manufacturer’s instructions. Each sample was run in triplicate with GAPDH as the endogenous control.

Primers used for quantitative real-time PCR were (5′-3′):

CCC​TGG​GGA​ACA​CTA​CAT​TTT​G, GCC​AAT​TCC​TAG​TCT​GTC​CAC​TT for Arg-1; AAG​CAC​ACT​GGT​TTC​CAC​ACT, TGG​GTC​CCT​GCA​TAT​CCG​TT for TNF-α; TGT​GGG​CAT​CAA​TGG​ATT​TGG, ACA​CCA​TGT​ATT​CCG​GGT​CAA​T for human GAPDH.

### Traswell assay to detect cell migration

The induced M0 macrophages were inoculated into the lower chamber of Transwell inserts (Corning, 3,422), and HCC cells were inoculated into the upper insert chamber. The lower chamber contained DMEM supplemented with 10% FBS to induce cell migration, whereas the upper chamber did not contain FBS. The same concentrations of Sch B were added to the upper and lower chambers. After 24 h of incubation, the cells were fixed with 4% paraformaldehyde and stained with 1% crystal violet for 20 min. The cells remaining on the upper side of the Transwell inserts were wiped off. The cells that passed through the membrane were observed under a microscope, and representative images were obtained.

### Flow cytometry to detect cell apoptosis and cell cycle

HCC cells were seeded at 2 × 10^5^ cells/dish in 6 cm dishes and cultured overnight in complete culture medium. Medium (4 mL) containing different concentrations of Sch B (0, 20, and 40 μg/mL) was added for 48 h. Centrifuge at 2000 rpm for 5 min and remove the supernatant. Annexin V-FITC and propidium iodide (Llovet et al., 2021) reagents were added according to the Annexin V-FITC/PI Apoptosis Kit (Elabscience, E-CK-A211). After gentle vortexing, the cells were incubated at room temperature in the dark for 15–20 min, and flow cytometry detection was performed within 1 h.

The treated HCC cells were collected by centrifugation, washed with phosphate-buffered saline (PBS), centrifuged to remove the PBS, resuspended in pre-cooled PBS, and slowly dripped into pre-cooled anhydrous ethanol to obtain a final concentration of 70%. Fixed at 4°C overnight, centrifuge to remove 70% anhydrous ethanol, add PBS to clean once, remove PBS.PI staining was performed with Cell Cycle Detection Kit (Keygen, Jiangsu, China, KGA512), and the fluorescence expression of PA channel was detected after incubation for 20 min in the dark.

### Exosome extraction and identification

THP-1 cells were treated with PMA for 48 h and HCCLM3 cells were co-culture for 12 h.The culture base was replaced with DMEM complete medium containing 20 μg/mL Sch B (10% exosome-free FBS was added, which was obtained by centrifugation at 100,000 *g* at 4°C for 18 h) for 48 h. The supernatant was collected, and the exosomal precipitate was extracted by differential centrifugation. The exosome precipitate was extracted using differential centrifugation. Briefly, cell culture supernatants were centrifuged at 300 *g* for 10 min to pellet the cells, centrifuged at 2, 000 × g for 10 min to remove dead cells, and centrifuged at 10, 000 × g for 30 min to remove cell debris. The final supernatants were ultracentrifuged at 120, 000 × g for 90 min to obtain exosomes. The exosomes were stored at −80°C prior to use. All procedures were performed at 4°C. Alix and CD9 proteins were detected by western blottingting, and GAPDH was used as a negative control. The morphology of the exosomes was observed by transmission electron microscopy (TEM). The size of the exosomes was measured using a ZETASIZER Nano Series Nano- ZS (Malvern Instruments, Malvern, United Kingdom).

### Proteomic analysis

Sch B (20 μg/mL) was used to treat the macrophage and HCCLM3 cell co-culture system, and the exosome precipitates from the Sch B treatment group and the control group were collected and sent to Guangzhou Spectrum Biotechnology Co., Ltd. for protein spectrum analysis.

### Animal experiment

All animal experiments were performed in strict accordance with the guidelines and were approved by the Animal Care Committee of Qingyuan Hospital Affiliated to Guangzhou Medical University (LAEC-2024-025). Male BALB/c nude mice were obtained from GemPharmatech (Guangzhou, China) for the establishment of the animal model. A subcutaneous injection of HCCLM3 cell suspension (100 µL) containing 2 × 10^6^ cells was administered into the left axilla of each mouse. On the third day after the subcutaneous inoculation of tumor cells, the mice were divided into two groups: a control group and a treatment group, with six mice in each group. Mice in the treatment group were intragastrically administered 100 mg/kg Sch B, whereas the control group was administered the same volume of the control solvent. After administering the drugs daily, the mice were euthanized samples were collected on day 30, and the subcutaneous tumors were removed to measure tumor volume and weight.

### Immunohistochemistry and immunofluorescence

The tumor samples were fixed with formaldehyde, embedded in paraffin, and sectioned (3.5 μm). Dewaxing was performed in xylene and graded from ethanol to water. The slides were immersed in EDTA solution (pH 9.0) and heated. After heating, the samples were kept at room temperature until they cooled to room temperature. A peroxidase scavenger was added and incubated to remove endogenous peroxidase from the tissue. After washing with PBS, an appropriate amount of primary antibody (Ki67: ZSGB-BIO, Beijing, China, TA800648) was added to the slides and incubated at 4°C overnight. After washing with PBS, the secondary antibody was added and DAB was used for color development. Finally, hematoxylin was used for counterstaining.

After the tumor samples were dewaxed to water as in the immunohistochemistry step, an appropriate amount of primary antibody (FN1: abcam, ab268020) was added to the slides, incubated overnight at 4°C, washed with PBS. Added fluorescent secondary antibody to react in the dark for 1 h, washed with PBS, and DAPI was added for sealing.

### Statistical analysis

Graphpad prism 8.0 software was used for data analysis. One-way ANOVA was used to analyze the experimental data between multiple groups, and the t-test was used to compare the experimental data between two groups. Each experimental group was repeated three times independently, and the quantitative data were expressed as mean standard deviation. Statistical differences were considered when p < 0.05.

## Results

### Sch B inhibits the growth of HCC cells

**FIGURE 1:**
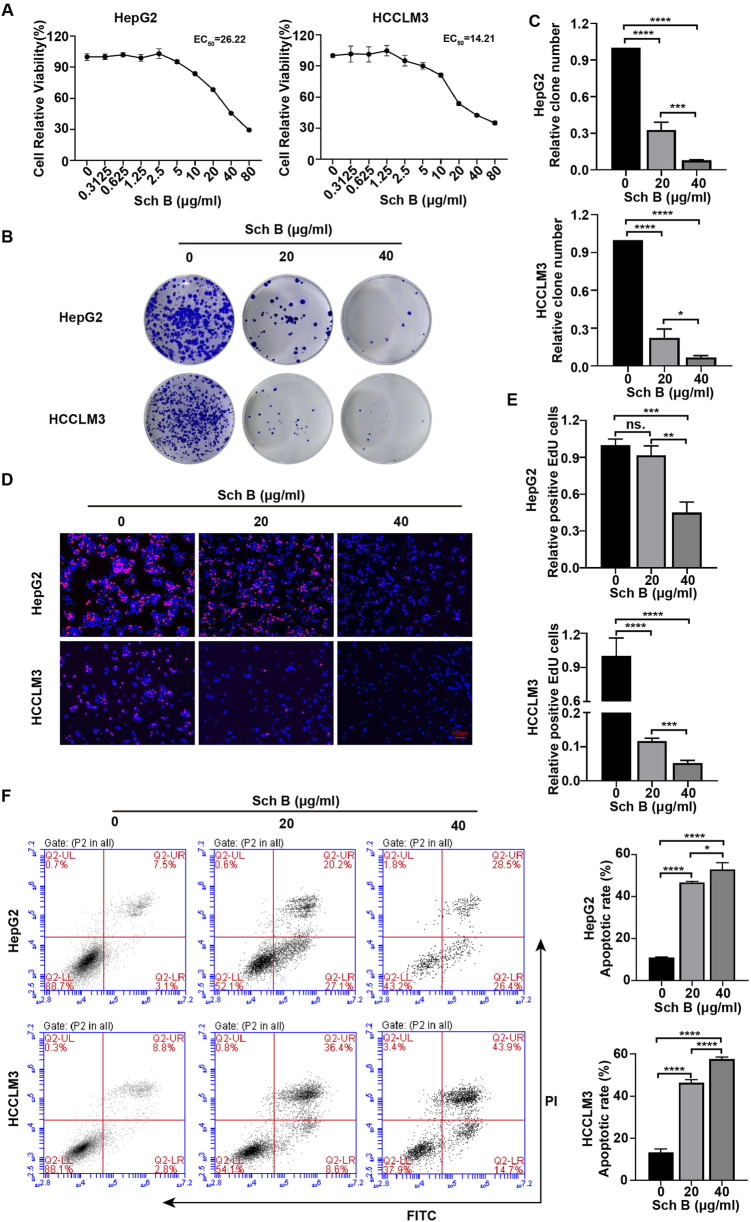
Sch B inhibits HCC cell growth. **(A)** HCC cells were treated with various concentrations of Sch B, and cell viability was measured using the CCK-8 assay. **(B–F)** HCC cells were treated with Sch B (0, 20 and 40 μg/mL), and their proliferation abilities were measured using plate cloning experiment (BC) and a EdU assay (DE). HCC cell apoptosis was detected using flow cytometry **(F)**. There sults are presented as the mean ± SD (n = 3). ns, not significant; *p < 0.05, **p < 0.01, ***p < 0.001, and ****p < 0.0001.

The CCK-8 method was used to assess the effect of Sch B on HCC cell lines. As the concentration of Sch B increased, the proliferation ability of HepG2 and HCCLM3 cells decreased, and their EC50 values were 26.22 μg/mL and 14.21 μg/mL, respectively (Figure 1A), indicating that Sch B inhibited the proliferation of HepG2 and HCCLM3 cells in a dose-dependent manner. EdU or 5-ethyl-2′-deoxyuracil is an analog of uracil, which can be incorporated into newly synthesized DNA to detect cell proliferation. With the increase in Sch B concentration, numbers of EdU-positive cells were significantly reduced and the difference was statistically significant (Figures 1D, E). In addition, Sch B inhibited the colony-forming ability of HepG2 and HCCLM3 cells (Figures 1B, C). To further assess the effect of Sch B on HCC cells, we showed that after Sch B acted on HCC cells, the apoptosis level of HCC cells increased in a dose-dependent manner compared to that of the control group (Figure 1F). These results indicate that Sch B inhibits HCC cell growth by promoting apoptosis and inhibiting proliferation.

### Sch B inhibits the growth of HCC cells in co-culture system

**FIGURE 2:**
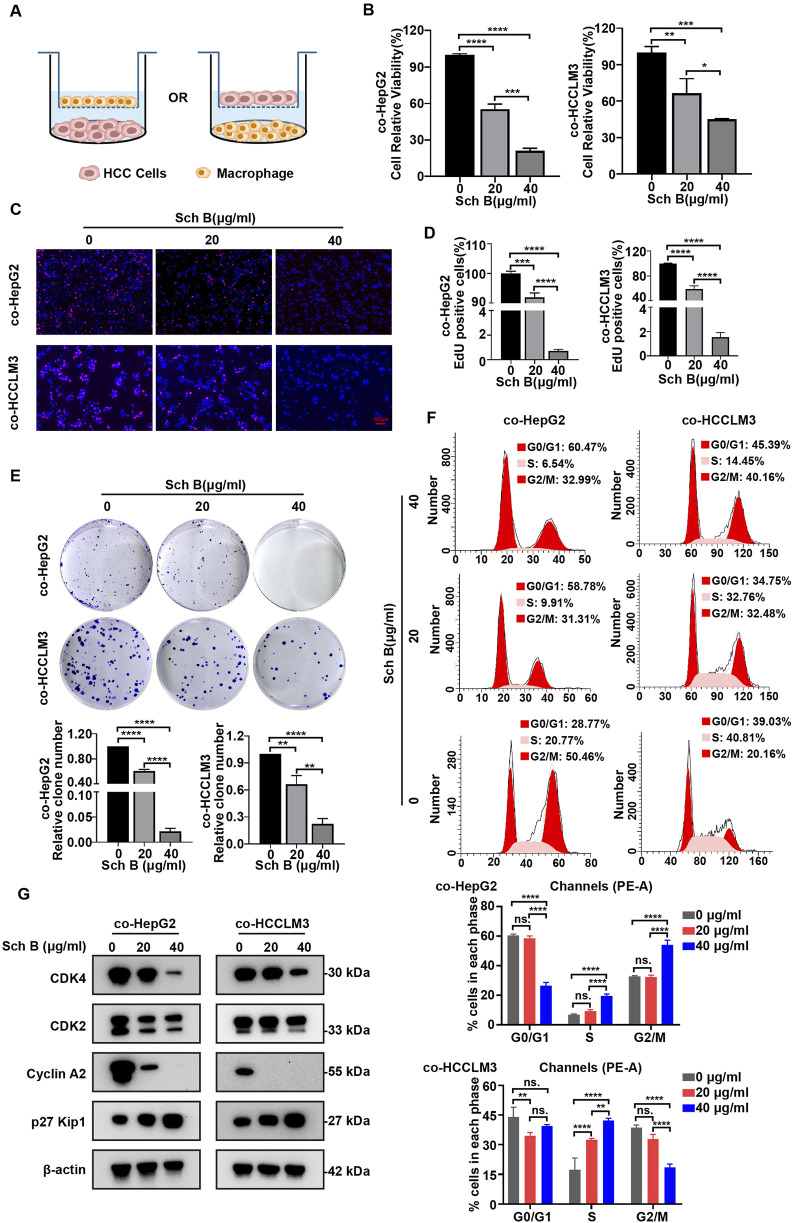
Sch B inhibits HCC cell growth in the co-culture system of macrophages and liver cancer cells. **(A)** Schematic diagram of the co-culture of HCC cells and macrophages. Sch B (0, 20, or 40 μg/mL) acted on the co-culture system, and HCC cell proliferation in the co-culture system was detected using cell counting **(B)**, the EdU assay **(C and D)** and the colony formation assay **(E)**. **(F)** Flow cytometry was used to assess the cell cycle and perform quantitative analysis of HCC cells in the co-culture system. **(G)** Western blotting was performed to measure CDK4, CDK2, cyclin A2, and p27 Kip1 protein expressions in HCC cells in the co-culture system. The results are presented as the mean ± SD (n = 3). *p < 0.05, **p < 0.01, ***p < 0.001, and ****p < 0.0001.

In the TME, macrophages account for approximately 50% of the immune cells and play an important role in tumor development (Lu et al., 2019). The interaction between macrophages and tumor cells may affect the antitumor effects of drugs (Lim et al., 2023). Therefore, we established a system of interaction between tumor cells and macrophages to explore the effects of Sch B on HCC cells. First, we established a co-culture system for HCC cells and macrophages. PMA-treated THP-1 or HCC cells were inoculated into the upper chamber of the Transwell inserts, and HCC cells or PMA-treated THP-1 cells were inoculated into the lower chamber for interaction (Figure 2A). During the interaction, the cells in the upper and lower chambers did not directly contact each other and communicated between cells through fluid exchange. The effect of Sch B on the viability of HCC cells in a co-culture system was investigated. As shown in the results of Figure 2B, Sch B inhibited the proliferation of HCC cells in the co-culture system. The same trend was observed in the EdU assay (Figures 2C, D). Moreover, Sch B inhibited the colony-forming ability of HCC cells in the co-culture system (Figure 2E). In addition, cell cycle analysis of HepG2 and HCCLM3 cells in the co-culture system showed that Sch B significantly arrested cells in S phase (Figure 2F). To further understand the regulatory proteins involved in cell cycle arrest, we analyzed the proteins of cell cycle checkpoints, including CDK4, CDK2, cyclin A2, and p27 Kip1, using Western blotting. The results showed that Sch B inhibited the protein expression of CDK4, CDK2, and cyclin A2 and upregulated the protein expression level of p27 Kip1 (Figure 2G). This suggests that Sch B affects the growth of HCC cells by regulating the cell cycle and inhibiting cell proliferation in a co-culture system.

### Sch B inhibits the migration of HCC cells in co-culture system

**FIGURE 3:**
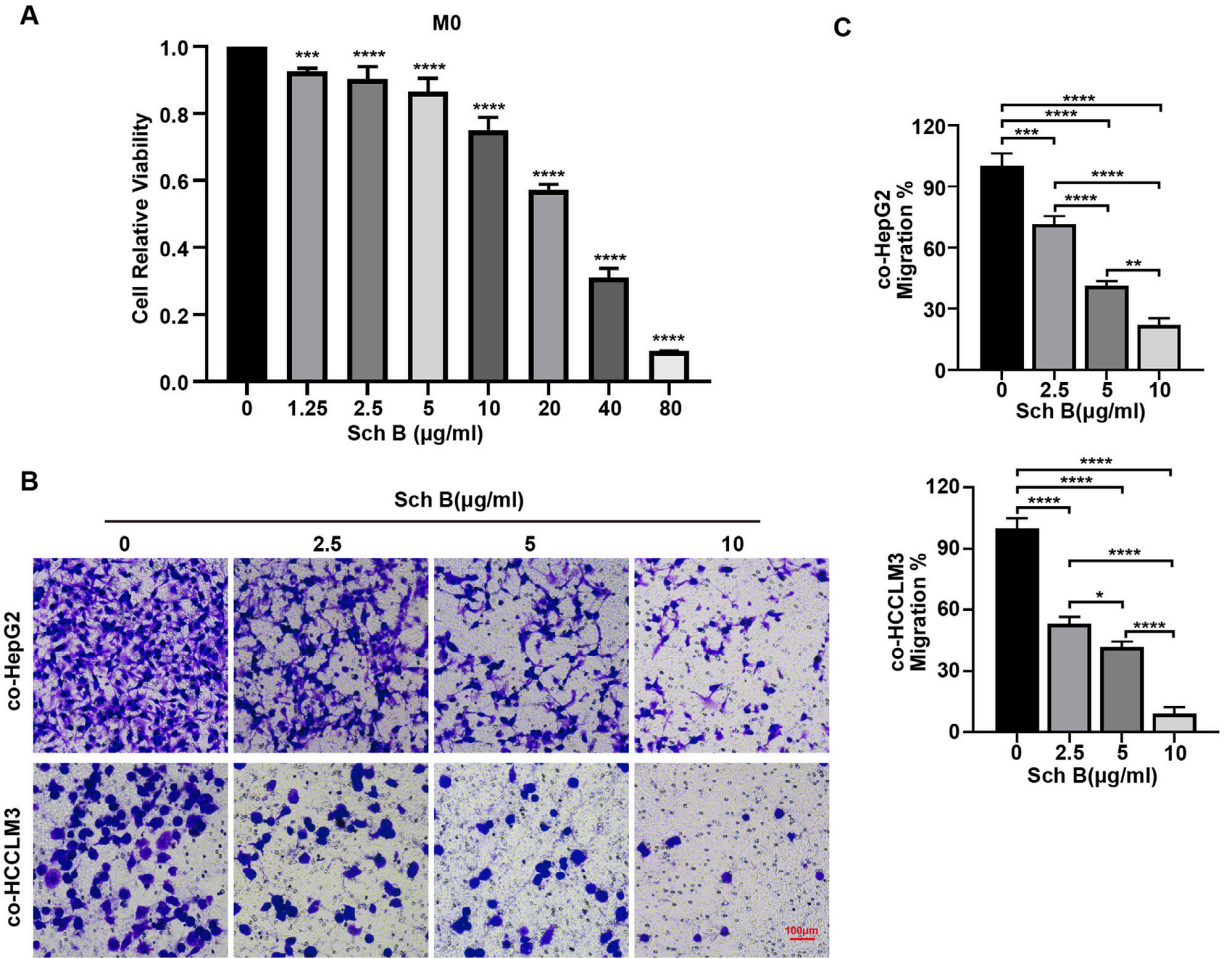
Sch B inhibits HCC cell migration in the co-culture system of macrophages and HCC cells. **(A)** M0 macrophages were treated with various concentrations of Sch B, and their cell viability was assessed using the CCK-8 assay. Sch B at concentrations of 0, 20, and 40 μg/mL was selected to treat the co-culture system, and the migration ability of HCC cells in the co-culture system was detected using the Transwell migration assay **(B)** and quantitative analysis **(C)**.

From the previous results, it was concluded that Sch B could affect the proliferative ability of HepG2 and HCCLM3 cells in the co-culture system, and whether Sch B could affect their migration ability was investigated. Therefore, the migratory ability of HCC cells in the co-culture system was tested. The viability of Sch B-treated M0 macrophages was detected using the CCK-8 assay (Figure 3A). To exclude the influence of Sch B on the proliferation ability of HCC cells and macrophages on the migration experiment, the Sch B-treated co-culture system with little effect on the cell viability of HCC cells and M0 macrophages was selected according to the results of Figures 1A, 3A, which were 0, 2.5, 5, and 10 μg/mL. Next, the Transwell migration assay was performed, and the results showed that with the increase of Sch B concentration, the number of HCC cells in the co-culture system migrating and perforating gradually decreased, with a statistically significant difference (p < 0.05) (Figures 3B,C). This indicated that Sch B inhibited the migration of HCC cells in the co-culture system.

### Extraction and identification of exosomes in Sch B-treated co-culture system

**FIGURE 4:**
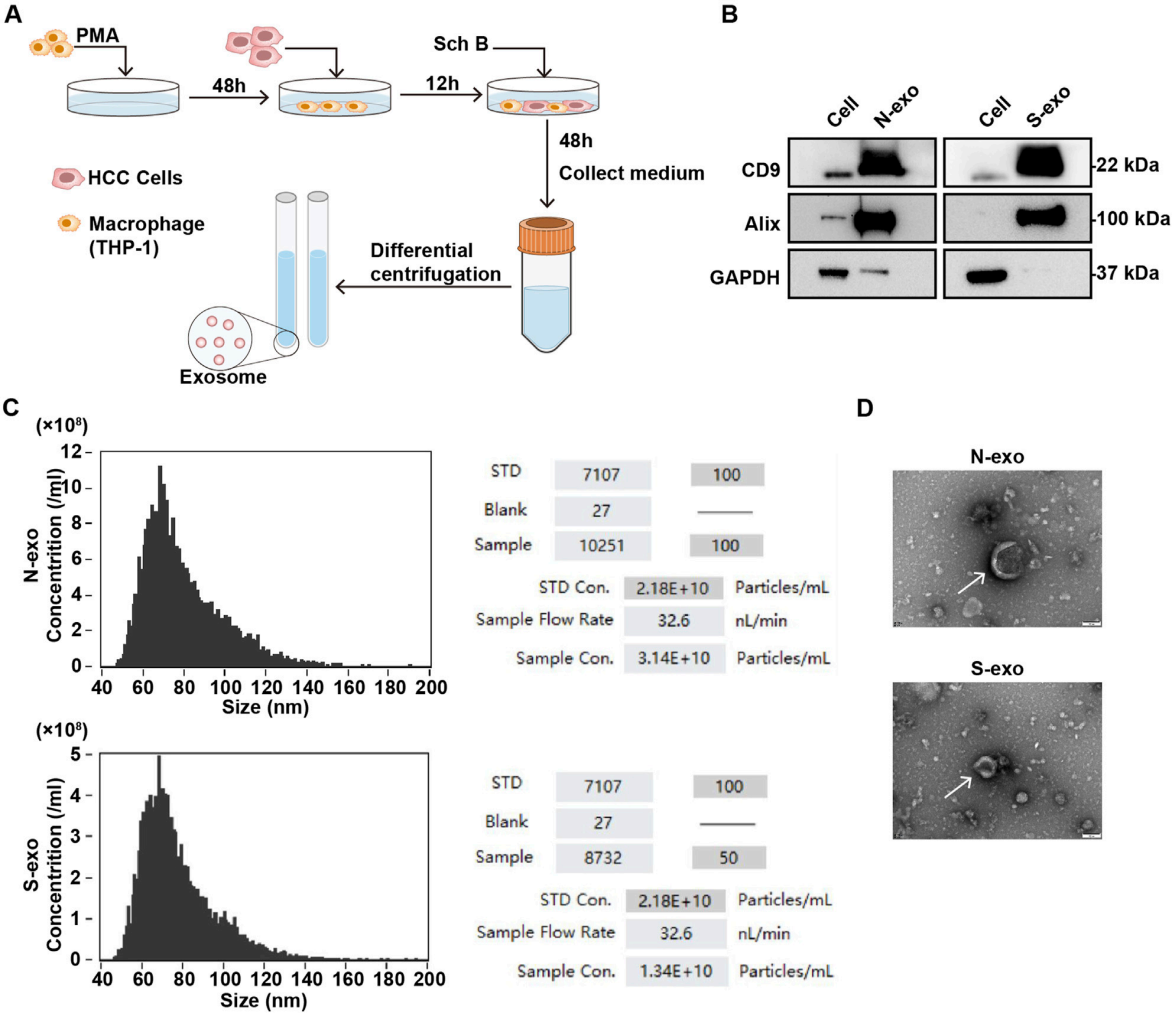
Identification of exosomes in the co-culture system with Sch B. **(A)** Sch B-treated (0 and 20 μg/mL) co-culture system with experimental flow diagram for collecting exosomes. **(B)** Western blotting was performed to detect the expression of exosome surface marker proteins CD9 and Alix, and GAPDH was used as a negative control. **(C)** Particle size analysis of exosome diameters and concentrations. **(D)** TEM analysis of exosome morphology. N-exo represents the exosome of the control group and S-exo represents the exosome of the Sch B-treated group.

Exosomes promote information exchange during cell interactions and are important mediators of celluar interactions in the TME. Therefore, we extracted exosomes using an interactive system for experimental identification. Macrophages were obtained after inducing THP-1 with PMA for 48 h. HCCLM3 cells were inoculated for interaction. After 12 h, Sch B (20 μg/mL) was added for 48 h. The supernatant was collected, and precipitated particles were obtained by ultra-high-speed gradient centrifugation (Figure 4A). The expression of the exosome markers Alix and CD9 proteins in the collected precipitated particles was detected, and GAPDH protein was used as a negative control. As shown in the Figure 4B, compared to the cell group, the collected pellet group highly expressed Alix and CD9 proteins. Particle size analysis revealed precipitate particles ranging in diameter of 50–120 nm (Figure 4C), which is within the particle size range of the exosomes. Compared with the control group, the concentration of exosomes in the Sch B-treated group decreased significantly (Figure 4C). The morphology observed using a TEM was round and vesicle-like (Figure 4D). These results suggest that the collected precipitated particles were exosome precipitates.

### SchB affects the regulation of M1 polarization by exosome FN1

**FIGURE 5:**
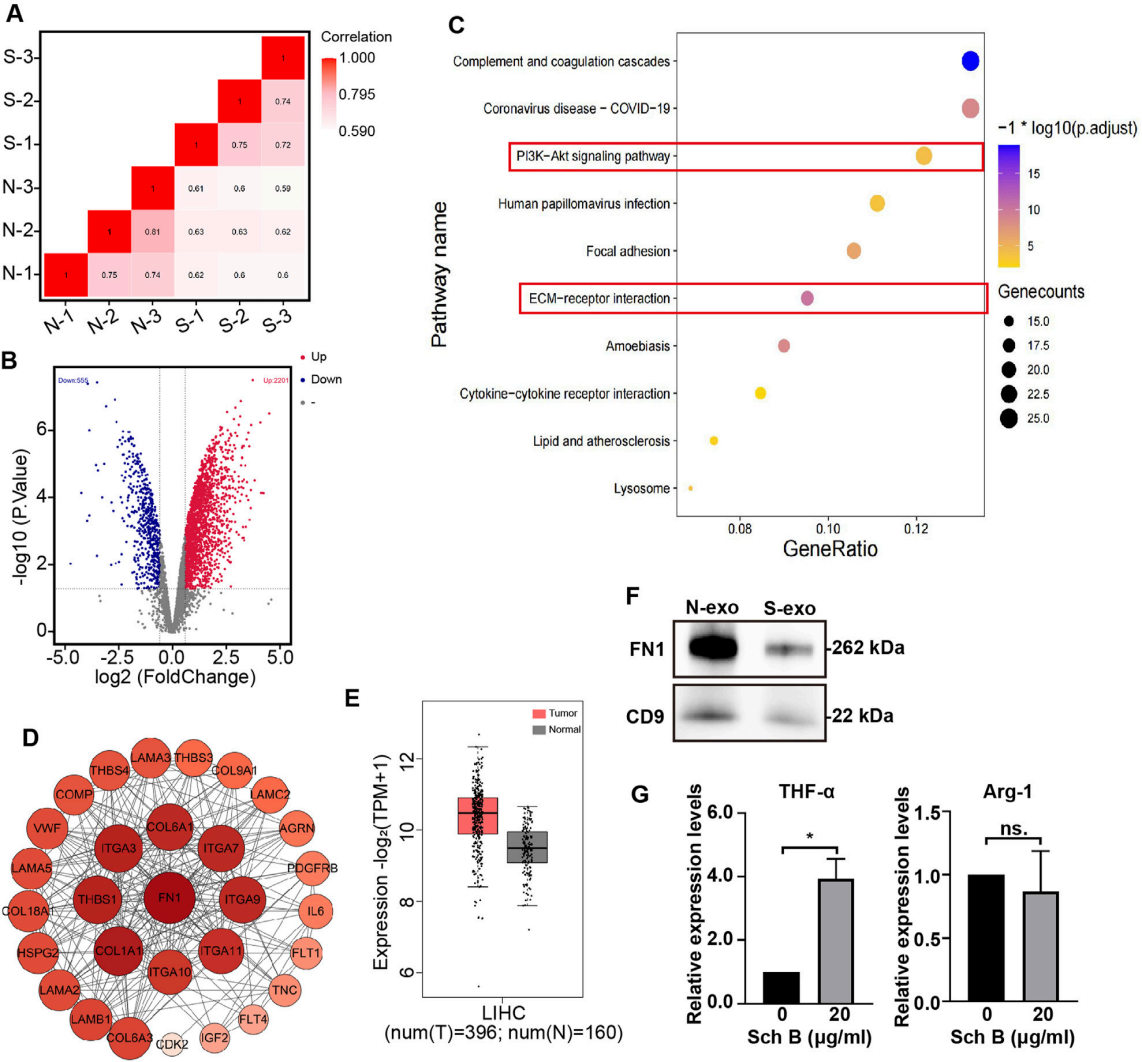
Proteomic analysis of exosomes in the Sch B-treated co-culture system. Exosome protein correlation analysis **(A)** and differential protein volcano map **(B)** of the control (N) and Sch B-treated (S) groups. **(C)** Bubble map of KEGG pathway enrichment analysis of downregulated differential proteins. **(D)** PPI analysis network map of differential proteins: the size and color depth of each circle represent the degrees. **(E)** Box plot of differential expression analysis of FN1 in patients with LIHC and healthy individuals. ****p < 0.0001. **(F)** Western blotting was performed to detect the expression of FN1 protein in exosomes. N-exo represents the exosome of the control group and S-exo represents the exosome of the Sch B-treated group (Sch B treatment concentration, 20 μg/mL). **(G)** Expression levels of TNF-α and Arg-1 in M0 macrophages in co-culture system. *p < 0.01.

Previous studies have shown that Sch B affects HCC cells in a co-culture system, but it is still unclear whether exosomes, an important communication tool in the TME, are affected by Sch B to regulate the proliferation and migration of HCC cells. Protein spectrum analysis was performed on exosomes in the co-culture system with no-Sch B treatment (N group) and Sch B treatment (20 μg/mL) (S group). The correlation between the N and S groups was high (Figure 5A), and 5,019 proteins were identified. Protein differential analysis was performed on the identified proteins, and the absolute fold change cutoff value (FC) was ≥1.5 and the p < 0.05. The results showed that 2,756 proteins were differentially expressed in the S and N groups, of which 555 proteins were lower in the S group than in the N group, and 2,201 proteins were significantly higher in those in the N group (Figure 5B). Previous studies have shown that Sch B affects the proliferation and migration of HCC cells in co-culture systems. Therefore, we focused on the signaling pathways that were more strongly correlated with cell proliferation and migration. Among the signaling pathways enriched by downregulated differentially expressed proteins, the PI3K-Akt signaling pathway and the ECM-receptor interaction pathway were related to cell proliferation and migration (Figure 5C). The differentially expressed proteins in these two pathways were subjected to protein-protein interaction networks (PPI) analysis. As shown in Figure 5D, the FN1 protein had the strongest interaction among the differential proteins involved in the PI3K-Akt signaling pathway and the ECM-receptor interaction pathway. Thus, FN1 plays an important role in liver cancer. Differential expression analysis of FN1 in patients with liver cancer and healthy individuals on the GEPIA 2 website showed that compared with normal people, FN1 expression was increased in patients with liver hepatocellular carcinoma (LIHC), *p* < 0.0001 (Figure 5E). Western blotting confirmed that the expression of exosomal FN1 in the Sch B-treated group was lower than that in the control group (Figure 5F). Macrophage-derived FN1 promotes the migration of liver cancer cells via the JUN pathway (Zhang et al., 2022). Moreover, the expression of FN1 was correlated with the polarization of M2 macrophages (Zhou et al., 2022). The levels of TNF-α and Arg-1 in M0 macrophages in the co-culture system were detected, and the results showed that the expression level of TNF-α was increased in the Sch B-treated group (Figure 5G). These results suggest that Sch B inhibits the proliferation and migration of HCC cells by regulating FN1 expression in cell-interacting exosomes, which may be related to M1 polarization.

### Sch B inhibits the expression of FN1 in HCC

**FIGURE 6:**
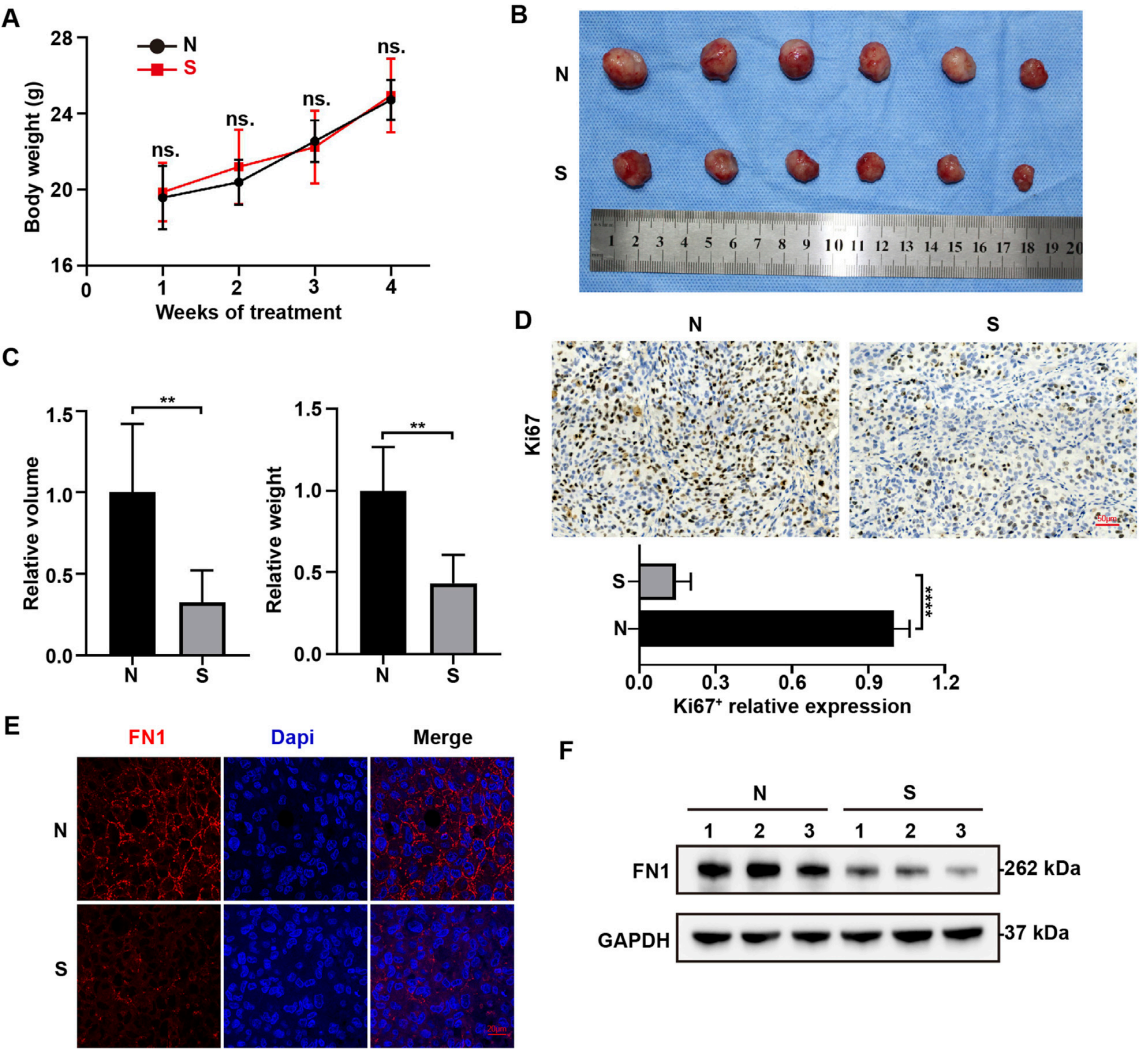
Sch B inhibits FN1 expression in HCC cells. Subcutaneous tumor formation in nude mice. **(A)** Body weights of mice in different treatment groups. **(B)** Tumor images of the different treatment groups. **(C)** Tumor volumes and tumor weight. **(D)** Immunohistochemistry was performed to assess Ki67 expression. Immunofluorescence **(E)** and Western blotting **(F)** were used to detect FN1 expression in tumors. **p < 0.01, ****p < 0.0001. N represents the control group and S represents the Sch B-treated group.

To further explore the effect of Sch B on HCC, HCCLM3 cells were subcutaneously injected into the armpits of nude mice and Sch B was administered by gavage at a concentration of 100 mg/kg. The control group was administered the same volume of control solvent. The body weight remained unchanged between the control group and the Sch B-treated group (Figure 6A). Compared with the control group, the tumors in the Sch B-treated group were smaller than those in the control group (Figure 6B). Tumor volume and weight (Figure 6C) decreased. The tumors were subjected to immunohistochemistry experiments, and the results showed that compared to the control group, the expression of Ki67 in the Sch B-treated group was reduced (Figure 6D), suggesting that Sch B can inhibit the growth of HCC tumors. Immunofluorescence analysis revealed that the expression of FN1 in the Sch B-treated group was lower than that in the control group (Figure 6E), and the same results were obtained in the Western blotting test (Figure 6F), suggesting that Sch B may inhibit the growth of HCC by inhibiting the expression of FN1.

## Discussion

Liver cancer is the most common fatal malignant tumor with a poor prognosis. Long-term use of chemotherapeutic drugs such as sorafenib can cause side effects such as hypertension, leukopenia, nausea, vomiting, and dyspnea. Therefore, further research into effective methods for the treatment of liver cancer are required. Natural compounds extracted from herbs, animals, or natural materials have structures similar to those of chemical compounds and can enhance the efficacy of current drug treatment regimens without increasing host toxicity, rendering them more attractive than synthetic compounds. In recent years, herbal extracts and natural compounds isolated from traditional Chinese medicine (TCM) have been used to treat patients with liver cancer. For example, the polyphenol curcumin extracted from turmeric rhizomes has antitumor, antioxidant, and anti-inflammatory effects, as well as various pharmacological effects on HCC cells (Darvesh et al., 2012). The active ingredient Sch B, extracted from the Chinese herbal medicine Schisandra chinensis, has been shown to have significant anti-inflammatory, antioxidant and cancer-inhibiting effects. Moreover, no notable effect on the cell viability of LO2 cells has been observed, and inhibits the occurrence and development of liver cancer *in vivo* and *in vitro* (Song et al., 2023; Yang and Wu, 2023). We verified that Sch B has an inhibitory effect on the proliferation of liver cancer cells through CCK-8, EdU, and colony formation experiments and showed that Sch B can promote the apoptosis of liver cancer cells. Therefore, Sch B may be an effective drug for the treatment of liver cancer.

The TME is an environment in which tumors survive, and is a complex system in which immune and stromal cells interact with cancer cells. The TME interacts with tumor cells to affect their growth (Sas et al., 2022). In addition to tumor cells, other immune cells can be found in the TME. Macrophages, which account for approximately half of all cells other than tumor cells, play a vital role in the occurrence and development of HCC (Lu et al., 2019). Under normal physiological conditions, macrophages act as “scavengers,” which regulate immune responses against pathogens and maintain tissue homeostasis. However, during the evolution of cancer, the TME affects tumor metabolism by directly exchanging metabolites and actively reprograms the metabolic processes of macrophages through cytokines and other signal transduction mediators (Mehla and Singh, 2019), thus promoting or inhibiting the occurrence and development of tumors. During treatment, it is important to understand whether the drug affects tumor cells in the macrophage-tumor cell interaction system. Exploring the mechanism of interaction between macrophages and cancer cells in the TME can help in tumor treatment. Previous studies have shown that Sch B can inhibit the growth of liver cancer cells; however, the effect of Sch B on the interaction between macrophages and liver cancer cells remain unknown. Therefore, an in-depth exploration of its role and mechanism of action is of great significance for the treatment of liver cancer. Therefore, the present study simulated the TME in which liver cancer cells and macrophages interact, and established a co-culture system between macrophages and HCC cells *in vitro* to explore whether Sch B has an effect on liver cancer cells in the co-culture system and its mechanism of action. Therefore, the purpose of this study was to provide novel insights into the use of the natural compound Sch B in liver cancer treatment.

We treated the interaction system of macrophages and HCC cells with Sch B and determined that Sch B inhibited the viability, DNA synthesis, and colony formation ability of HCC cells in a dose-dependent manner. Cell cycle dysregulation is the primary cause of liver cancer cell proliferation. Our results showed that Sch B primarily blocked HCC cells in the S phase in the co-culture system. The cell cycle process is primarily mediated by cyclin-dependent kinases (Bisteau et al., 2014). Analysis of cell cycle checkpoint proteins in HCC cells in the co-culture system showed that the expression of CDK4, CDK2, and cyclin A2 was reduced, whereas the expression of the cyclin-dependent kinase inhibitor p27 Kip1 was increased, indicating that Sch B may inhibit the growth of liver cancer by regulating the cell cycle of HCC cells in the co-culture system. In addition, at concentrations that had little effect on the viability of macrophages or HCC cells, Sch B inhibited the migration of HCC cells in the co-culture system in a dose-dependent manner. This indicates that Sch B inhibits the proliferation and migration of HCC cells in a co-culture system.

In the TME, exosomes carry various proteins, growth factors, RNA, and DNA that promote cell communication. For example, the HCC cell-derived exosome pyruvate kinase M2 (PKM2) promotes HCC progression by inducing macrophage polarization, thereby reprogramming the TME and promoting HCC (Hou et al., 2020). Previous experimental results have shown that Sch B can inhibit the proliferation and migration of HCC cells during the interaction between macrophages and HCC cells; however, whether it affects the production of exosomes during this interaction is still unclear. Therefore, the extracellular vesicles from the co-culture system were collected in this study. The collected extracellular vesicles were identified as exosomes by detecting the expression of the exosome marker proteins Alix and CD9, particle size analysis, and morphology analysis using TEM. The concentration of collected exosomes decreased after the addition of Sch B. Proteomics was used to analyze protein changes in exosomes in the co-culture system with or without Sch B intervention. The results showed that after the addition of Sch B, 555 proteins were downregulated and 2,201 proteins were upregulated. Previous studies have shown that Sch B inhibited the proliferation and migration of HCC cells in a co-culture system. To this end, we performed KEGG signaling pathway enrichment analysis on significantly downregulated differentially expressed proteins, among which the enriched pathways were significantly related to cell proliferation and migration, including the PI3K-Akt signaling pathway and the ECM-receptor interaction pathway. Previous studies have shown that the PI3K-Akt signaling pathway can promote the proliferation of tumor cells, and thus promote tumor (He et al., 2021). The ECM is a key component of the TME that supports tumorigenesis, and its disorder is a prominent feature of cancer. Increased ECM cross-linking in the TME increases the stiffness of the pathological microenvironment, and the hardening of the ECM stimulates tumor growth and migration (Jiang et al., 2022). Therefore, the inhibition of HCC cell proliferation and migration in the co-culture system by Sch B may be related to the downregulation of the PI3K-Akt signaling and ECM-receptor interaction pathways. Further PPI analysis of the differentially expressed proteins enriched in the PI3K-Akt signaling and ECM-receptor interaction pathways showed that FN1 had the strongest interaction among all the differentially expressed proteins. FN1 promotes the proliferation of tumor cells and tumor metastasis by regulating the EMT (Li et al., 2019; Wang et al., 2021; Tan et al., 2021). In addition, FN1 deposition creates a favorable microenvironment that promotes HCC metastasis (Liu et al., 2024; Lopez-Canovas et al., 2023). Differential expression analysis of FN1 showed that FN1 was highly expressed in patients with liver cancer compared to normal individuals. Furthermore, we verified the expression of exosomal FN1 in a co-culture system with and without Sch B intervention. The results showed that after Sch B treatment, the expression of FN1 protein in exosomes decreased. Studies have shown that overexpression of FN1 can promote the polarization of M2 macrophages (Zhou et al., 2022). We examined the expression of macrophage markers in the co-culture system. The results showed that the expression of TNF-α (M1 macrophage marker) was significantly upregulated in the Sch B treatment group, while the expression of Arg-1 (M2 macrophage markerr) was not significantly changed. It is worth noting that this phenomenon is apparently inconsistent with the anti-inflammatory properties of Sch B itself, suggesting that Sch B may induce the polarization of macrophages towards pro-inflammatory M1 phenotype by reprogramming the immune microenvironment under the specific regulation of the TME. This suggests that Sch B may inhibit the proliferation and migration of HCC cells by inhibiting FN1 protein in the exosomes of the co-culture system, which may be related to the polarization of M1 macrophages. To further verify the effect of Sch B on HCC, we conducted an *in vivo* experiment and showed that Sch B inhibited the growth of HCC tumors, and the expression of FN1 in the Sch B-treated group was lower than that in the control group. In addition, *in vivo* experimental results also verified the conclusion that Sch B inhibits the growth of liver cancer by inhibiting the expression of FN1.

Overall, the present study verified that Sch B could inhibit the proliferation and migration of HCC cells in the macrophage-HCC cell interaction system. This process may be the result of Sch B inhibiting the production of exosomes in the macrophage-HCC cell interaction system and downregulating exosomal FN1 protein expression. In addition, Sch B inhibit the expression FN1 thereby inhibit HCC growth. However, the molecular mechanism though which Sch B downregulates FN1 to exert its anti-HCC effects remains unclear and requires further exploration and verification.

### Limitations of the study

Although our study provides new insights into the anti-tumor effects of natural compounds Sch B in the HCC microenvironment, there are still many limitations. Firstly, in our study, the exosomal FN1 protein was derived from the co-culture system of macrophages and HCC cells, while the source of FN1 protein was not distinguished. Additionally, we detected that Sch B may play an anti-tumor role by down-regulating FN1 protein, but the specific regulatory mechanism is not yet clear. Moreover, our study relied on xenograft mouse models to explore the inhibitory effect of Sch B on HCC, but clinical sample verification was lacking. Future studies should focus on the specific regulatory mechanism of FN1 by Sch B and the detection of clinical models.

## Data Availability

The original contributions presented in the study are included in the article/supplementary material, further inquiries can be directed to the corresponding authors.
